# Fungal biological control agents for integrated management of *Culicoides* spp. (Diptera: Ceratopogonidae) of livestock

**DOI:** 10.14202/vetworld.2015.156-163

**Published:** 2015-02-10

**Authors:** B. W. Narladkar, P. R. Shivpuje, P. C. Harke

**Affiliations:** 1 Department of Veterinary Parasitology, College of Veterinary and Animal Sciences, Maharashtra Animal and Fishery Sciences University, Parbhani, Maharashtra, India; 2 Department of Agricultural Entomology, Marathwada Agricultural University, Parbhani, Maharashtra, India; 3 Microbiology unit under DBT research Project, Department of Veterinary Parasitology, College of Veterinary and Animal Sciences, Maharashtra Animal and Fishery Sciences University, Parbhani- Maharashtra, India

**Keywords:** biocontrol agents, *Beauveria bassiana*, *Culicoides* spp, integrated pest management, *Metarhizium anisopliae*

## Abstract

**Aim:**

Entomopathogenic fungi *Metarhizium anisopliae* and *Beauveria bassiana* had wide host range against insects and hence these are being exploited as fungal bio-pesticide on a large scale. Both fungi are proved pesticides against many crop pests and farmers are well acquainted with their use on the field. Thus, research was aimed to explore the potency of these fungal spores against larval and adult *Culicoides* midges, a pest of livestock.

**Materials and Methods:**

*In-vitro* testing of both fungal biological control agents was undertaken in Petri dishes against field collected *Culicoides* larvae, while in plastic beakers against field collected blood-engorged female *Culicoides* midges. *In-vivo* testing was undertaken by spraying requisite concentration of fungal spores on the drainage channel against larvae and resting sites of adult *Culicoides* midges in the cattle shed. Lethal concentration 50 (LC_50_) values and regression equations were drawn by following probit analysis using SPSS statistical computerized program.

**Results:**

The results of this study revealed LC_50_ values of 2692 mg and 3837 mg (10^8^ cfu/g) for *B. bassiana* and *M. anisopliae*, respectively, against *Culicoides* spp. larvae. Death of *Culicoides* larvae due to *B. bassiana* showed greenish coloration in the middle of the body with head and tail showed intense blackish changes, while infection of *M. anisopliae* resulted in death of *Culicoides* larvae with greenish and blackish coloration of body along with total destruction, followed by desquamation of intestinal channel. The death of adult *Culicoides* midges were caused by both the fungi and after death growth of fungus were very well observed on the dead cadavers proving the efficacy of the fungus.

**Conclusion:**

Preliminary trials with both funguses (*M. anisopliae, B. bassiana*) showed encouraging results against larvae and adults of *Culicoides* spp. Hence, it was ascertained that, these two fungal molecules can form a part of biological control and alternative to chemical control and, therefore, can be inducted in integrated management programs.

## Introduction

Using chemical/synthetic pesticides as a single tactic in controlling an important and significant livestock pests have been proved as dangerous as their indiscriminate use have often resulted in problems such as pesticide resistance, pest resurgence, residual toxicity, imbalance in ecological equilibrium, etc. To overcome these problems against any insect pest a strategy involving integrated management in the form of modules is being planned in the present era. Particularly, the integrated pest management (IPM) modules formed against crop pests have yielded tremendous results. However, such IPM modules against livestock pests are yet a dream. Therefore, undertaking the research for development of different biological control agents (BCAs) against important pests of livestock, for inducting in IPM programs, is the need of an hour. Efficacy of two funguses *Beauveria bassiana* and *Metarhizium anisopliae* against adult and larvae of houseflies *Musca domestica* [1-3]; against *Haematobia irritans* [4] and against mosquito larvae [5-7] is well-documented. The fungus *M. anisopliae* (ICIPE-30) and *B. bassiana* (IMI-391510) spores proved as efficacious in infecting and killing larvae of *Anopheles stephensi* and *Anopheles* *gambiae* under laboratory conditions [8]. It has been demonstrated that infection of adult mosquitoes *Culex quinquefasciatus* with *B. bassiana* causes a significant reduction in their survival and disease transmission under field conditions [9].

However, very few studies were undertaken against the *Culicoides* midges [10,11] and particularly no report is available from India on efficacy of both these funguses against *Culicoides* midges. *Culicoides* spp. midges are painful biters, blood suckers and responsible for transmission of many viral diseases of livestock commonly occurring in India. In view of the above-mentioned facts, attempts were made for exploring the potency of these two proven entomopathogenic fungi against *Culicoides* spp. larvae and adult stages.

## Materials and Methods

### Ethical approval

Since prime objective of the study was to develop the fungal candidates as biological control agents for integrated management of *Culicoides* midges, for which evaluation of two fungal candidates against larvae in drainage channels and against adult midges on the wall of cattle shed were proposed. The institutional animal ethical committee approved the proposed study ensuring that no potential harm toward animal welfare and no direct application of fungus on animal body which will result in any discomfort to the animals.

### *In-vitro* testing of fungal BCAs

Two fungal candidates were evaluated against field collected larvae of *Culicoides peregrinus* by adapting the techniques described by Busvine [12]. 25 ml test solution prepared from requisite powder of fungal spores, as mentioned in Table-1, was added in each Petri dish. For undertaking *in-vitro* tests, water used for making dilutions of fugal bio-pesticides were from the same drainage channel, where from the larvae were collected. It was used after harvesting complete larvae and decantation. The idea behind using such water was that, whatever microbial flora and fauna available as food for larvae at natural sites shall be available in petri-dishes too. 25 *Culicoides* larvae were transferred to each of the test Petri dishes with the aid of pasture pipette. *Culicoides* larvae were collected from natural breeding sites as per the standard method of larval harvesting [13] by using a simple technique of sedimentation. Mortality was observed after 24, 48, 72, 96 and 120 h of treatment. Experiment was conducted with six replications. Lethal concentration 50 (LC_50_) values and regression equations were drawn by following probit analysis using SPSS statistical computerized program.

**Table-1:** *In-vitro* and *in-vivo* testing of fungal BCAs against field collected *Culicoides* larvae.

Concentration g/100 ml	Number of trials^#^	Mortality percentage in petri dish after hours post treatment (Mt)			Mortality percentage in petri dish after hours post treatment (Bv)		
	
		24	48	72	24	48	72
0.1	05	0.0±0.0	0.0±0.0	0.0±0.0	0.0±00	0.0±00	0.0±00
0.2	05	0.0±0.0	0.0±0.0	0.0±0.0	20.0±0.98 (18-22)	20.0±0.98 (18-22)	20.0±0.98 (18-22)
0.4	05	20.0±0.98 (18-22)	20.0±0.98 (18-22)	20.0±0.98 (18-22)	64.67±1.33 (60-72)	68.33±1.33 (64-72)	68.33±1.33 (64-72)
0.5	05	67.83±3.53 (48-80)	81.33±0.56 (80-84)	81.33±0.56 (80-84)	75.88±0.85 (72-80)	83.33±2.44 (75-96)	83.33±2.44 (75-96)

	**Day PT^$^**	**Larval count per 100 g of mud/drainage (control)**		**Larval count per 100 g of mud/drainage (Mt)**		**Larval count per 100 g of mud/drainage (Bv)**	

Field trial with 5.0 g of fungal powder per liter of water+5.0 ml of milk sprayed @ 30 ml/m^2^ area of drainage channel	0	87.33±4.56 (75-100)		149.16±6.73 (130-165)		105.00±7.63 (80-130)	
	1	76.00±1.16 (72-80)		149.16±6.73 (130-165)		105.00±7.63 (80-130)	
	2	83.33±2.44 (75-96)		47.00±5.65 (20-50)		50.00±0.85 (50-64)	
	3	81.33±0.56 (80-84)		72.00±5.35 (50-80)		66.33±2.04 (60-76)	
	4	81.33±0.56 (80-84)		20.00±0.56 (15-22)		75.88±0.85 (72-80)	
	5	95.33±3.65 (80-130)		81.00±0.56 (80-84)		22.88±3.85 (16-42)	
	6	95.33±3.65 (80-130)		31.33±5.6 (20-50)		83.33±2.44 (75-96)	
	7	145.00±8.65 (130-160)		81.00±0.56 (80-84)		70.00±2.04 (65-84)	
	8	Larval count showed increasing trend		Resuming the population of larvae towards as on day 0		Resuming the population of larvae towards as on day 0	

# Each trial conducted with six replicates (n=30 [6×5]),

$ Average of six trials conducted at different periods on same drainage channel with same BCA, PT: Posttreatment, BCA: Biological control agents

### *In-vivo* trials of BCAs

Trials were undertaken by spraying fungal solution of desired concentration on drainage channels, around the livestock shed, which were positive for larvae of *Culicoides* spp. Each drainage channel was labeled with nameplate for a particular fungus and concentration. Each drainage channel used for spraying fungus solution was selected for 10 m length and then sealed at both proximal and distal ends. The purpose of sealing was to cease the new addition of larvae accruing from incoming water from the proximal end of the channel. Larval count was recorded before and after spraying at 24 h interval till larval count reaches negligible or zero level. Larval count was recorded by using the standard method of larval harvesting [13] in a weighed quantity of substrate collected from the drainage channel.

### *In-vitro* and *in-vivo* testing against adult C. peregrinus midges

Trials were conducted as:

- Trial A (*In-vitro*): Trials of each fungus were conducted in plastic beakers of 1000 ml volume (10 cm diameter and 15 cm height). Filter paper treated with dried fungal powder @ 750 mg/900 cm (30 cm^2^ × 30 cm^2^) area was adhered to sides and bottom of the beaker, in which 25 blood engorged females were released, and it was closed with muslin cloth with the aid of rubber band with one beaker as control. Beakers were maintained in the desiccators having RH (75%) in the laboratory having room temperature of maximum ranging between 29.4°C and 33.3°C and minimum ranging between 13.9°C and 22.4°C during the study period. Beakers were daily observed for mortality of midges and cadavers of died midges were collected with Camlin hair brush for further processing
- Trial B (*In-vivo*): For this experiment, sites selected were inner walls of cattle shed. On these walls *Culicoides*, midges rests after blood engorgement. Both fungus in the solution form prepared by mixing 5 g powder+ 5 ml milk + 1000 ml of water, applied on marked square of 2 × 2 feet (60 cm × 60 cm) @ 30 ml/m^2^ area. *Culicoides* midges resting on such sites were collected with the help of insect aspirator and were maintained in the laboratory for observing the mortality. Dead cadavers of midges from treated and control squares were incubated for fungal growth. Each observation was replicated 6 times.

### Processing of dead midges

To wipe out the fungal spores adhered to the body of dead cadavers of *Culicoides* midges collected from trials A and B, were washed with several rinsing of normal water. Such cadavers were rolled in the moist tissue paper and kept in desiccators having humidity level of 75% at room temperature. The humidity levels were created by adding NaCl solution (40 g/100 ml of water) at the bottom of desiccators. Dead cadavers of *Culicoides* were observed for growth of fungus under zoom stereoscope daily for more than 15 days.

## Results and Discussion

### Effect of fungus on larvae and adult *Culicoides* midges in vitro and in vivo

**Figure-1:**
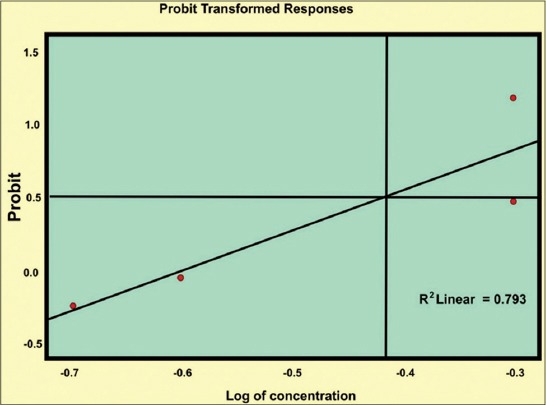
Probit line for *Metarhizium anisopliae* against *Culicoides* larvae.

**Figure-2:**
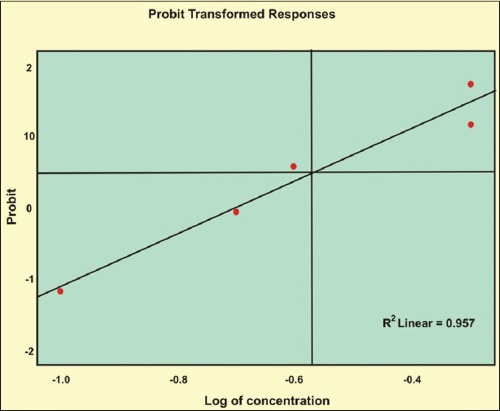
Probit line for *Beauveria bassiana* against *Culicoides* larvae.

**Figure-3:**
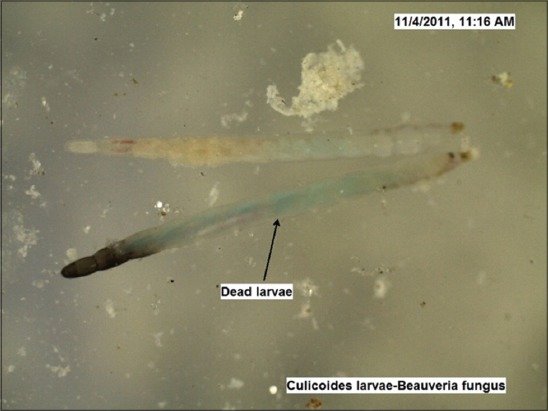
*Culicoides* larvae died due to *Beauveria bassiana* infection (×50).

**Figure-4:**
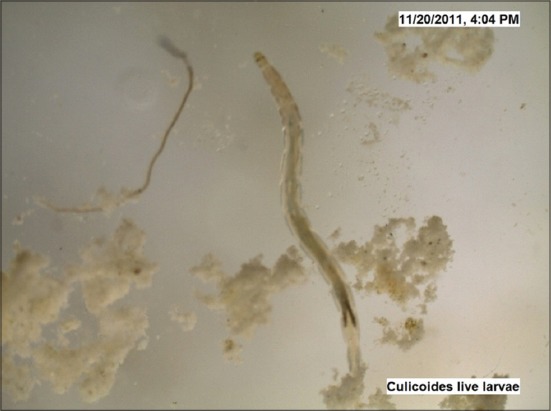
Normal cadaver of *Culicoides* larvae (control) (×50).

**Figure-5:**
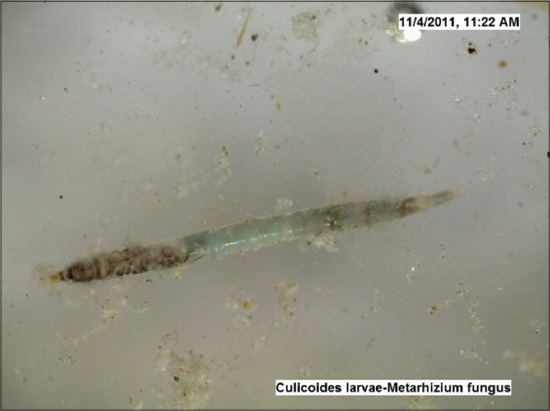
*Culicoides* larvae died due to *Metarhizium anisopliae* (×50).

**Figure-6:**
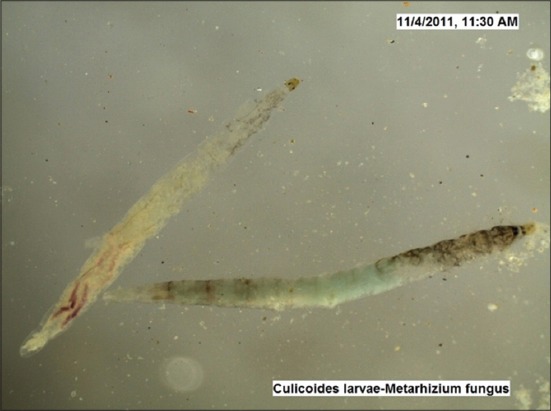
*Culicoides* larvae died due to *Metarhizium anisopliae* (×50).

The LC_50_ values estimated against *Culicoides* larvae were 2692 mg and 3837 mg (10^8^ cfu/g) for *B. bassiana* and *M. anisopliae*, respectively by drawing the probit lines (Tables-2 and 3; Figures-1 and 2). Data from Table-1 indicated that fungal infection to *Culicoides* larvae resulted in mortality and therefore larval count was consistently showing decreasing trend, although, daily there was the addition of newer larvae hatched from eggs laid by *Culicoides* midges. However, 7 days post infection, the larval count was resuming to its original number as observed on day 0 and both funguses found effective for maximum 7 days, necessitating its innudative application. Death of *Culicoides* larvae due to infection of *B. bassiana* showed greenish coloration in the middle of the body, while head and tail showed intense blackish changes (Figure-3) against normal body texture of larvae (Figure-4). Infection of *M. anisopliae* resulted in death of *Culicoides* larvae with greenish and blackish coloration of body (Figures-5 and 6) along with total destruction and desquamation of intestinal channel.

**Table-2:** LC_50_ values of different bio-pesticides used against *Culicoides* larvae and its comparison with LD_50_ values in rats.

BCA	LC_50_ mg/L	LD_50_ value in rats	Remarks in context to LD_50_
*B. bassiana* (Bv)*	2692 mg (10^8^ cfu/g)	*B. bassiana* strain HF23 at a dose of 4.05×10^9^ cfu/animal [28]. *B. bassiana* strains ATCC-74040 [29]: Acute, toxicity and pathogenicity: Oral infectivity rat: LD50>1.9×10^8^ cfu/animal; no evidence of toxicity, pathogenicity, or infectivity. Spores still detectable in faeces on day 14. Acute intratracheal/inhalation toxicity in rat: LD_50_>2.5×10^9^ cfu/animal; initial body weight decrease, local reversible signs of lung inflammation. No evidence of pathogenicity or infectivity. Clearance in lung tissue completed by day 15 postdosing. Acute intravenous/intraperitoneal toxicity in rat: LD_50_>2.0×10^7^ cfu; no evidence of toxicity (nasal secretion on day 1), pathogenicity or infectivity. Clearance in blood completed by day 2 postdosing	On the basis of (a) LD_50_ values and evaluation studies of various strains of these two funguses which proved them as potent against dipterans pests [1-11,28-30], (b) extensive use of two funguses, against crop pests, and (c) thus, well acquaintance of farmers with these funguses, present study recommends their use in IPM program against *Culicoides* spp.
*M. anisopliae* (Mt)*	3837 mg (10^8^ cfu/g)	LD_50_ acute oral, rats>2000 mg/kg; LD_50_ acute dermal, rats>2000 mg/kg, intraperitoneal injection, rat tolerated (5×10^9^ spores/kg bodyweight), LC_50_ acute inhalation toxicity 4.85 mg, skin irritation, rabbit nonirritant [30]	

* Source=Powder containing 10^8^ conidia/g was procured from Entomology Department, Agricultural College, Mahatma Phule Agricultural University, Rahuri Dist Ahemednagar (MS) India, LC_50_=Lethal concentration 50, LD_50_=Lethal dosage, BCA=Biological control agents, *B. bassiana=Beauveria bassiana, M. anisopliae=Metarhizium anisopliae*

**Table-3:** Probit regression equations for fungal BCAs against *Culicoides* spp larvae.

BCA	Regression equation	Fiducial limit		

		Lower	Upper
Bv	PROBIT (p)=Intercept (2.694)+ B (3.866)×X (log concentration)	2.824	4.908
Mt	PROBIT (p)=Intercept (1.975)+ B (3.582)×X (log concentration)	2.504	4.660

BCA=Biological control agent

**Figure-7:**
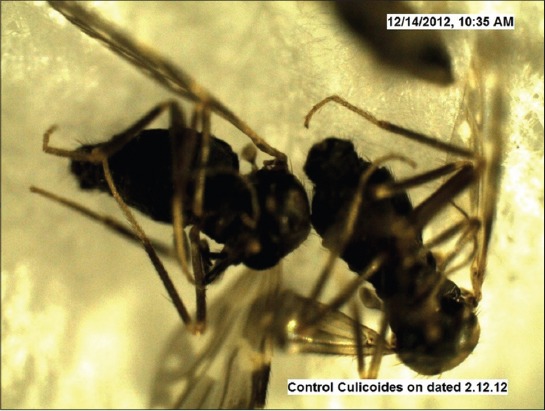
*Culicoides peregrinus* normal cadaver (control) on day 11 *PI* (×50).

**Figure-8:**
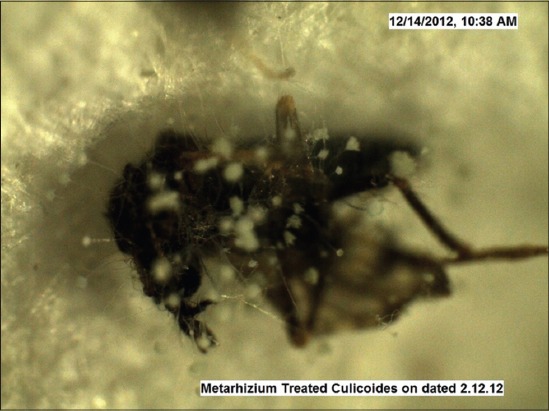
*Culicoides peregrinus* growth of *Metarhizium anisopliae* on day 11 *PI* (×50).

**Figure-9:**
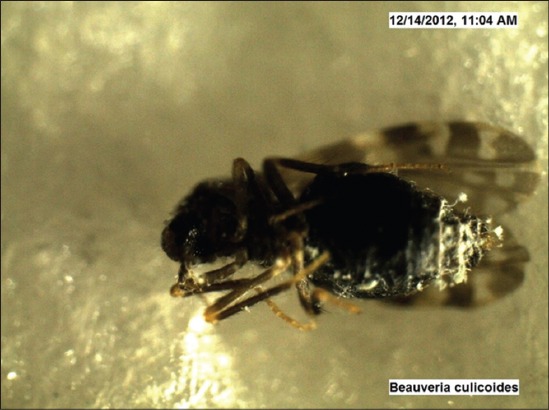
*Culicoides peregrinus* growth of *Beauveria bassiana* on day 11 *PI* (×50).

**Figure-10:**
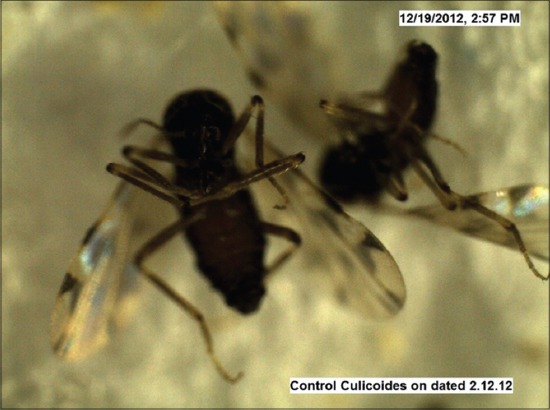
*Culicoides peregrinus* normal cadaver (control) on day 17 *PI* (×50).

**Figure-11:**
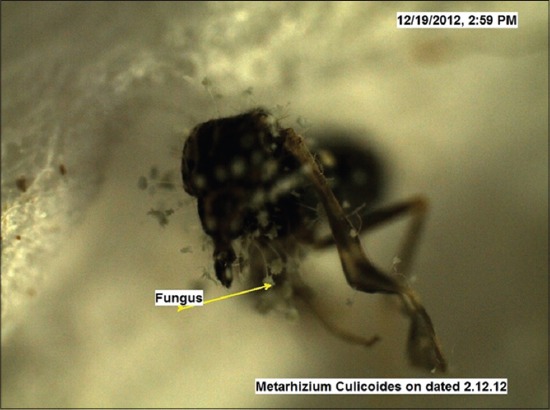
*Culicoides peregrinus* growth of *Metarhizium anisopliae* on day 17 *PI* (×50).

**Figure-12:**
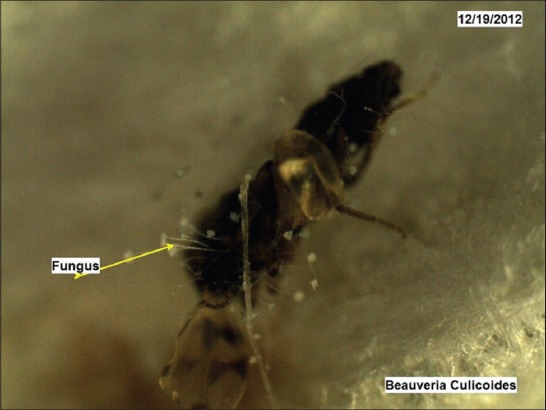
*Culicoides peregrinus* growth of *Beauveria bassiana* on day 17 *PI* (×50).

Under trial “A” death of adult *Culicoides* midges was caused by both the fungi within 24-48 h, while death of midges in the control was observed after 96 h and after death growth of fungus was very well observed on the dead cadavers of infected midges (Figures-7-12). Close look to Figures-7 and 10 indicated that up to 11-17 days post infection, cadavers in the control group were without any growth of fungi, contrary to it, on day 11 (Figures-8 and 9) and on day 17 post infection (Figures-11 and 12) fungal treated cadavers showed extensive fungal growth. Results of trial “B” revealed that, midges collected from treated square died within 24-48 h against 96 h in control and dead cadavers from treated groups, upon incubation for 8-15 days at room temperature and 75% RH showed tremendous growth of fungus, proving that both fungi holds the capacity to produce infection at the existing humidity levels. However, on large scale simulated studies could not be undertaken, because after catching infection by midges, when they fly, further fate of infection cannot be ascertained.

The purpose of the present study was not to compare the efficacy of two fungi studied and data was not generated to that context, but grossly it can be opined that, under prevailing environmental conditions of Marathwada (Parbhani) region of Maharashtra state, *M. anisopliae* fungus found comparatively better choice in terms of larval mortality and growth of fungus in the body of adult midges. Place of present study, Marathwada region lies between Latitude 17°35’N-20°40’N Longitudes 70°40’-78°15’ E, MSL - 40.9 m, in the Deccan Plateau Zone, is basically an intense agrarian region. Climatologically it was categorized as semi-arid on an annual basis. It is humid to per-humid during monsoon, sub-humid to semi-arid during winter and arid during the summer season. The corresponding distribution pattern of annual rainfall (500-1100 mm) is 75-85, 10-15 and 6-10%, respectively. The wet monsoon (weeks 23-44, 4^th^ June-4^th^ November) period alternates with long rain free cold winter (weeks 45-9, 5^th^ November-4^th^ March). Minimum temperature ranges between 12°C and 18°C and maximum between 40°C and 45°C is observed during the summer (weeks 10-22, 5^th^ March-3^rd^ June), having appreciable temporal and spatial variability, a typical characteristics of semi-arid climate [14].

Entomopathogenic fungi are widely distributed throughout the fungal kingdom, although the majority of them occur in the *Deuteromycotina* and *Zygomycotina* group of which *M. anisopliae* and *B. bassiana* has wide host range and hence these are being exploited as fungal bio-pesticide on large scale. The mode of action on generalized line of fungus against insects was narrated by Goettel and Inglis [15]. According to their study, most common route of host invasion is through the external integument (contact with conidiospores, i.e., conidia). Though, infection via digestive tract is possible. Conidia attach to the cuticle, germinate, and penetrate the cuticle with the help of enzymatic degradation and pressure of the germ tube [16,17]. Once in the hemocoel, the mycelium grows throughout the host, forming hyphal bodies called blastospores. Death of the insect is often due to a combination of action of fungal toxins, physical obstruction of blood circulation, nutrient depletion and/or invasion of organs. After the host dies, hyphae usually emerge from the cadaver and under suitable abiotical condition, conidia are produced on the exterior of the host. These are then dispersed by wind or water. The infection may also take place via the respiratory system [18]. After infection, free-floating, yeast-like hyphal bodies are produced and spread throughout the hemocoel [19]. Similarly, data from Table-3 also indicates that LC_50_ values of both fungi and their corresponding LD_50_ values are indicative of their safety and therefore can be recommended for induction in IPM programs.

It will be not be out of context to review mode of action and efficacy of both fungi judged by various authors against the mosquito larvae as detailed by Scholte *et al*. [20] in reference to present study.

#### B. bassiana

Conidial dry powder when poured on water surface of breeding sites of mosquito, conidia being hydrophobic, thus floating on the water surface, and contact mosquito larvae that feed below the surface mainly at the tip of the siphon, blocks the respiration and thereby kills the larvae. In adults conidia enters through the spiracles, get germinated, invades the walls of the tracheae, and subsequently were thought to release a toxin that kills the adults [21]. Subsequent studies about the toxins of this genus confirmed were beauvericin, bassianin, bassianolide, beauverolides, and tenellin from *B. bassiana*, [22]. Hart and MacLeod [23], who recorded the germination of *Beauveria* conidia described the requirement as RH above 94%, however infection does not appear to be dependent on temperature [16]. In laboratory tests against adult *Culex tarsalis, Culex pipiens*, *Aedes aegypti, Ochlerotatus sierrensis, Ochlerotatus nigromaculis* (Ludlow), and *Anopheles albimanus*, conidia of *B. bassiana* produced 100% mortality within 5 days after exposure [21]. Sections of infected adults, fixed and imbedded in paraffin immediately after death, revealed some mycelium in regions surrounding the main tracheal trunks. From these observations, [21] inferred that the conidia appeared to be entered through the spiracles, germinated, invaded the walls of the tracheae, and subsequently were thought to release a toxin that killed the adults.

In the light of these observations, it can be stated that fungus infection studied against *Culicoides* midges appears to be resulted in producing similar type of pathogenesis. When fungus was applied at the resting sites of *Culicoides*, they picked up the infection, may be through tarsi, and death took place within 24 h with ample growth of fungus on the body after 10 days period (Figures-9 and 12). Thus, spray of *B. bassiana* fungus on resting sites of *Culicoides*, can be the effective way of biological control of adult *Culicoides* midges. *Beauveria* is one of the most frequently isolated entomogenous fungal genera and has a cosmopolitan distribution, though the genus has a very broad host range [24]. In three small-scale outdoor tests with conidia of *B. bassiana*, reductions of 82, 95 and 69% were found on *C. pipiens* larvae and pupae after 2 weeks [21]. Similar type of results was obtained in regards to reduction in *Culicoides* larvae when *B. bassiana* fungus was applied to drainage channels in the present field study (Table-1). Despite consistent results obtained in the lab, on-field results were vague and inconsistent. Two drawbacks of *B. bassiana* fungus mentioned in the literature are, (1) Susceptible species were prone to infection only shortly after moulting. If the infection occurred shortly before moulting, the mycelium was lost within the moult, (2) a problem associated with using conidia is that they have no residual effect. They germinate in mosquito habitats even when not in contact with larvae. In the present project field trials, too, it appeared that fungus needs innudative application without showing much residual effect (maximum up to 7 days).

#### M. anisopliae

According to Boucias and Pendland [25], fungus has a large host-range, including arachnids and five orders of insects. On terrestrial insects, the life cycle begins with conidia attaching to the host cuticle, forming an appressorium, followed by a penetration peg to enter the cuticle. After entering the hemocoel, hyphae formed that produces and releases toxins, killing the host within 4-16 days. These toxins released are destruxins, swainsinone, and cytochalasin C [22]. If the conditions are warm and moist, conidiophores will grow through the cuticle to cover the insect with conidia. At breeding sites, if conidia are applied on the water surface, larvae contact to conidia through perispiracular valves during their air intake. The fungus germinates and penetrates into the respiration siphon, blocking the breathing mechanism [26,27]. Plugging of the spiracles usually leads to death before significant invasion of the hemocoel occurs, so hyphal body formation is minimal. The optimal growth temperature for most strains is 27-28°C and RH 92% [16]. According to the literature, *M. anisopliae* has several characteristics that make it interesting as a microbial mosquito control agent. It has advantage that this do not germinate in the mosquito environment until actual exposure to a host and its resulting persistence in the environment, as well as the fact that its effect is not limited to periods of host moulting, which has taken this fungus up as compared to *B. bassiana*, and hence make this fungus a very promising control agent [24]. One drawback mentioned in the literature in regards to use of *M. anisopliae* for mosquito control is that conidia are not produced on submerged fungus killed larvae, and hence inundated releases are required. Perhaps this could be reason that conidia may not be getting produced on dead *Culicoides* larvae and hence in the present study too, innudative releases of fungus on drainage channels was felt essential after 7 days PI (Table-1).

## Conclusion

Efficacy of both the fungus *B. bassiana* and *M. anisopliae* were proved against adult and larvae of housefly, *M. domestica* (Diptera: Muscidae) [1,2] and against *H. irritans* [4], against mosquito larvae [5-7]. All these observations indicate that both fungus candidates are proved as efficacious against many livestock pests, with the addition of the present study indicating their efficacy against *Culicoides* adults/larvae. Thus, use of these two fungus candidates is the novel weapon in the hands of farmers for covering management of range of insect pests.

## Author’s Contributors

BWN being PI of the research project conceptualized and implemented the technical program. BWN collected, processed and analyzed the data and submitted it in the form of present research article. PRS being Co-PI was involved in the finalization of technical program and involved in validation and confirmation of data and taxonomy related work of adult and larval *Culicoides* midges. PCH, Junior research fellow in the project and specialized in Microbiology, carried all laboratory work related to fungus and also contributed for undertaking *In-vivo* and *In-vitro* trials. All authors read and approved the final manuscript.
